# Development and validation of an electronic database-based frailty index to predict mortality and hospitalization in a population-based study of adults with SARS-CoV-2

**DOI:** 10.3389/fmed.2023.1134377

**Published:** 2023-05-12

**Authors:** Paola Rebora, Carlo Alberto Scirè, Giuseppe Occhino, Francesco Bortolan, Olivia Leoni, Francesco Cideni, Alberto Zucchelli, Emanuele Focà, Alessandra Marengoni, Giuseppe Bellelli, Maria Grazia Valsecchi

**Affiliations:** ^1^Bicocca Center of Bioinformatics, Biostatistics and Bioimaging, School of Medicine and Surgery, University of Milano-Bicocca, Monza, Italy; ^2^School of Medicine and Surgery, University of Milano-Bicocca, Monza, Italy; ^3^Regione Lombardia, General Directorate for Welfare, Regional Epidemiological Observatory Organizational Unit, Directorate General for Health, Milan, Italy; ^4^Department of Clinical and Experimental Sciences, University of Brescia, Brescia, Italy; ^5^Division of Infectious and Tropical Diseases, Department of Clinical and Experimental Sciences, University of Brescia, Brescia, Italy; ^6^Acute Geriatric Unit San Gerardo Hospital, Monza, Italy

**Keywords:** care transitions, health services, public health, COVID-19, hospital, community

## Abstract

**Background:**

Electronic health databases are used to identify people at risk of poor outcomes. Using electronic regional health databases (e-RHD), we aimed to develop and validate a frailty index (FI), compare it with a clinically based FI, and assess its association with health outcomes in community-dwellers with SARS-CoV-2.

**Methods:**

Data retrieved from the Lombardy e-RHD were used to develop a 40-item FI (e-RHD-FI) in adults (i.e., aged ≥18 years) with a positive nasopharyngeal swab polymerase chain reaction test for SARS-CoV-2 by May 20, 2021. The considered deficits referred to the health status before SARS-CoV-2. The e-RHD-FI was validated against a clinically based FI (c-FI) obtained from a cohort of people hospitalized with COVID-19 and in-hospital mortality was evaluated. e-RHD-FI performance was evaluated to predict 30-day mortality, hospitalization, and 60-day COVID-19 WHO clinical progression scale, in Regional Health System beneficiaries with SARS-CoV-2.

**Results:**

We calculated the e-RHD-FI in 689,197 adults (51.9% females, median age 52 years). On the clinical cohort, e-RHD-FI correlated with c-FI and was significantly associated with in-hospital mortality. In a multivariable Cox model, adjusted for confounders, each 0.1-point increment of e-RHD-FI was associated with increased 30-day mortality (Hazard Ratio, HR 1.45, 99% Confidence Intervals, CI: 1.42–1.47), 30-day hospitalization (HR per 0.1-point increment = 1.47, 99%CI: 1.46–1.49), and WHO clinical progression scale (Odds Ratio = 1.84 of deteriorating by one category, 99%CI 1.80–1.87).

**Conclusion:**

The e-RHD-FI can predict 30-day mortality, 30-day hospitalization, and WHO clinical progression scale in a large population of community-dwellers with SARS-CoV-2 test positivity. Our findings support the need to assess frailty with e-RHD.

## 1. Background

The unprecedented burden on healthcare systems caused by the coronavirus disease 2019 (COVID-19) pandemic has highlighted the need for tools to assist decision-makers in planning resource allocation. Since the first wave of the pandemic, healthcare providers have focused on the need to promptly identifying risk factors for COVID-associated severe disease and death (1). Age and comorbidity have been widely adopted as the main, if not the only, criteria to allocate healthcare resources and stratify care levels in affected individuals (2, 3). However, these parameters do not fully capture the biological heterogeneity of individual health trajectories, especially in older patients. Indeed, there is significant variability in socio-economic, functional, and cognitive status across individuals, as well as in lifestyle habits.

Frailty is a clinical syndrome that can be defined as a decline in body functions and physiological reserves, resulting in increased vulnerability and reduced resilience to physical and mental stressors, ultimately leading to an increased risk of negative health outcomes (4). Several studies have shown that frailty is useful to detect the heterogeneity in risk among people of the same chronological age (5, 6), including middle-aged individuals (7–9). Frailty can also predict survival, even in the absence of comorbidity (7, 10). Based on this evidence, international guidelines recommend frailty screening in order to stratify patients according to their risk of poor outcomes (11, 12). Furthermore, screening for frailty at the population level is appropriate to identify those who would benefit from a comprehensive geriatric assessment (CGA). CGA can provide the basis for a coordinated and integrated intervention to maximize the overall health status of the individual (13).

Among the available operationalizations of frailty, the so-called Frailty Index (FI), proposed by Rockwood and colleagues, is one of the most used (14). The FI measures the accumulation of health deficits (i.e., clinical signs, symptoms, diseases, and disabilities) that tend to increase with aging. The FI is obtained by calculating the ratio between the number of deficits observed in a person and the total number of deficits considered for its computation (15, 16). In several studies, FI was associated with increased mortality risk in both hospitalized (6, 17–21) and community-dwelling COVID-19 patients (22).

However, few studies have used an automated FI, based on routine collection of administrative community health data (23, 24), and none used a similar approach during the COVID-19 pandemic. In this context, electronic regional health databases (e-RHDs) represent an important resource. E-RHDs could identify people at higher risk for severe outcome or death, even when detailed information, usually collected in clinical studies, is not available.

Therefore, we undertook this study with the following objectives:

- To identify health deficits in adult beneficiaries of the Lombardy Regional Health System, using an e-RHD.
- To develop and validate a frailty index based on an e-RHD (e-RHD-FI).
- To assess whether e-RHD-FI is an adequate source of information on frailty, compared to a clinically based frailty index (c-FI).
- To assess the association between e-RHD-FI and adverse health outcomes in adult beneficiaries of the Lombardy Regional Health System with SARS-CoV2.

## Methods

### Data source and population

#### Electronic-regional healthcare database

Patients who had a positive nasopharyngeal swab polymerase chain reaction (PCR) test for SARS-CoV-2 by May 20, 2021 (*n* = 821,475) were extracted from the Lombardy region COVID-19 database. Patients aged 18 years and older who were beneficiaries of the Lombardy Regional Health system on December 31, 2019 (*n* = 689,197, in the Supplementary Figure S2) were selected for the analyses. Fully anonymized patient data were retrieved from the database, from routine physician visits between 2017 and 2021. The following variables were included: demographics, outpatient care, inpatient care, emergency healthcare and transports, primary care, delivery of assistive products for people with disability, mental illness care, home care services, residential care, drug prescriptions, exemptions (available since 2010), and mortality. Data were collected on exemptions from healthcare payments for selected conditions.

### Identification of health deficit and determinants from the e-RHD

Health deficits and determinants were retrieved by cross-referencing the different data sources in the e-RHD. Health deficits were defined as symptoms, signs, disabilities, and diseases, covering a range of systems and domains (i.e., Physical, Mental and Social). Chronic diseases were derived from the algorithm used for the management of chronic patients in Lombardy (25, 26). The co-payment exemptions database, Integrated Home Assistance, and Intermediate Assistance Observation Sheet datasets were evaluated to obtain a proxy for socio-economic status and to identify nursing home residents. Physical disabilities were retrieved from exemptions, Integrated Home Assistance, and prosthesis delivery service. Mental illness data were used to assess the mental domain (27, 28). The algorithms used to select these variables were based on previously published studies (23, 24) and were validated in the community. Full details on data source structure, health deficits and determinants, and algorithm development are reported in Supplementary material, Frailty index derivation on Regional Healthcare Databases section.

### Electronic-regional healthcare database frailty index derivation

The Electronic-regional healthcare database frailty index (e-RHD-FI) was developed according to Rockwood’s model (16, 29) in adult beneficiaries of the Lombardy Regional Health System who had a positive nasopharyngeal swab PCR test for SARS-CoV-2. Health deficits and determinants were assessed prior to the COVID-19 diagnosis. The date of positivity at the nasopharyngeal swab test for SARS-CoV-2 was used as the index date. We applied a variable recall period of 1 to 10 years, according to different types of health deficits and determinants (see Supplementary Tables S1, S2 for more details). For each patient, a 40-items e-RHD-FI was calculated as the ratio between the sum of the observed health deficits and determinants and the total number of health deficits and determinants included in the index (theoretical range 0–1). All deficits contributed to the index with 1 point, except income, which was classified into three categories: upper-middle/high income (no contribution to the index), lower-middle income (0.5 contribution), and low income (1 point contribution).

### Clinical data used to validate the e-RHD-FI

The FRACOVID study is an observational multicentre study which included 1,344 COVID-19 patients admitted to 2 hospitals in Lombardy (6). The study recorded 36 variables including sociodemographic, biological, and clinical data, symptoms, and disabilities. Data were collected on admission to two acute Geriatrics and two Infectious Diseases wards at Spedali Civili Hospital in Brescia and San Gerardo Hospital in Monza (6). A clinical frailty index (c-FI) was calculated in accordance with Rockwood’s model (16). Furthermore, the Clinical Frailty Scale (CFS) was also assessed (14). Both c-FI and CFS were significantly associated with in-hospital and 1-year mortality (6). Data from the FRACOVID study were used to validate the e-RHD.

### Ethics approval

This study was approved by the Brianza Institutional Review Board under the number 3356–07/08/2020.

### Statistical analysis

#### Validation of the e-RHD-FI versus c-FI, and clinical outcome

To assess the concurrent criterion validity of the e-RHD-FI, we compared it with the c-FI and the CFS of patients included in the FRACOVID study (6). A probabilistic model was defined using information on sex, age at nasopharyngeal swab test positivity, and hospital records (dates of admission and discharge, mortality, and center) to link patients included in the FRACOVID study with their data from the e-RHD (30, 31). Further details on the model are available in Supplement (Probabilistic Matching section). Spearman’s correlation coefficient (rho) was used to test the correlation between e-RHD-FI, c-FI, and CSF. The performance of e-RHD-FI and c-FI for predicting in-hospital mortality was compared using the area under the Receiver Operating Characteristic (ROC) curve (AUC). The association between e-RHD-FI and in-hospital mortality was assessed in the matched dataset by a Cox regression model after adjustment for sex, age, and period of hospital admission.

#### Association between e-RHD-FI and adverse health outcomes among beneficiaries of the Lombardy regional health system with SARS-CoV-2

Different end-points were evaluated: (i) 30-day mortality; (ii) 30-day hospitalization; (iii) a composite endpoint of 30-day mortality or hospitalization; (iv) an ordinal end-point based on the WHO clinical progression scale for COVID-19 at 60 days after SARS-CoV-2 test positivity (i.e., non-hospitalized, hospitalized, admitted to intensive care unit (ICU) or dead). Mortality was ascertained from death registers. Hospital and/or ICU admission were obtained from the COVID-19 database and from hospital discharge records. We randomly split the cohort into a training set (70%) and a validation set (30%). We identified the optimal cut-off using the Youden index on the ROC curve in the training set. In the validation set, we estimated the AUC of the ROC curve. Survival was estimated by the Kaplan–Meier method, and differences between groups defined according to the optimal cut-off were tested using the log-rank test. To categorize frailty in four groups, we used the identified cut-offs, along with two other cut-offs used in previous studies; namely 0.13 (which is the cut-off used in the FRACOVID study) (6) and 0.25 (which is the cut-off used in most non-COVID studies) (32, 33). The observation time started from the date of the positive SARS-CoV-2 swab. All patients were followed up until death, emigration/end of reception of regional assistance or June 19, 2021, whichever occurred first. For the 30-day hospitalization endpoint and the composite endpoint, the observation time stopped at the time of hospitalization. Cause-specific Cox proportional hazards regression models were used to investigate the association between e-RHD-FI and time to event (30-day mortality, 30-day hospitalization, and the composite of both). Ordinal logistic regression was used to test the association between the e-RHD-FI and the WHO clinical progression scale. Potential confounders were sex, age, and period of the diagnosis (before or after July 1, 2020). The prognostic ability of the e-RHD-FI was also assessed in population subgroups. Participants were stratified according to age class, calendar period (according to COVID-19 waves), and hospitalization (hospital admission or not). Hazard Ratios (HRs) or Odds ratios (ORs) with 99% confidence intervals (CIs) were reported. All analyses were performed using SAS version 9.4 (SAS Institute Inc., Cary, NC). The alpha error was set at 0.01 due to the large sample size (two-tailed).

## Results

**Figure 1:**
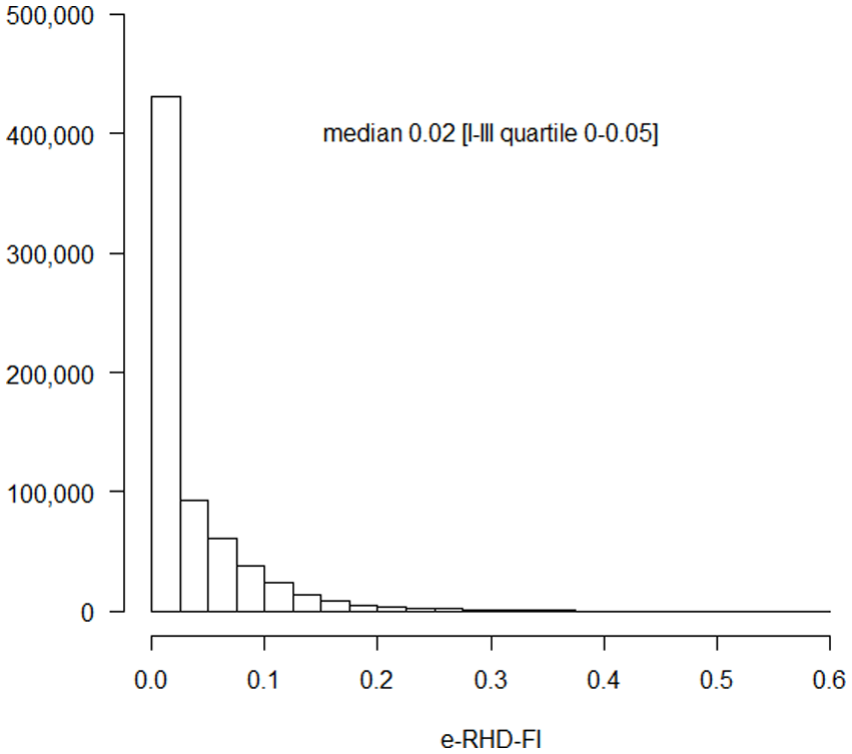
Regional Healthcare Database Frailty Index (RHD-FI) distribution in 689,197 adult subjects with SARS-CoV-2 up to 20th May 2021. The absolute frequency is reported on the vertical axis.

A total of 689,197 adult beneficiaries of the Lombardy Regional Health System (357,495 females, 51.9%) were positive at a nasopharyngeal swab PCR test for SARS-CoV-2 by May 20, 2021 Supplementary Figure S2. The median age was 52 years (first-third quartile, 38–66) and the Charlson Comorbidity Index (CCI) (34) was 0 for 96% of the population. The health deficits and determinants included in the e-RHD-FI are reported in Table 1 along with their prevalence. The most frequent deficit/determinant was hypertension (156,560, 22.7%) followed by low income (116,097, 16.8%) and heart disease (90,655, 13.2%). The e-RHD-FI showed a typical asymmetric distribution (Figure 1) with a median of 0.02 (first-third quartile, 0–0.05).

**Table 1:** Variables included in the electronic Regional Healthcare Database Frailty Index (e-RHD-FI) and their prevalence in 689,197 adults with SARS-CoV-2 up to 20 May 2021.

Deficit ID	Description	*N* (%)
FC_001	Respiratory failure/Oxygen therapy	1,176 (0.2)
FC_009	Arthritis and related disorders	37,746 (5.5)
FC_012	Cerebrovascular disease	24,271 (3.5)
FC_014	Chronic obstructive pulmonary disease and allied conditions	26,667 (3.9)
FC_017	Contusion with intact skin surface	46,112 (6.7)
FC_019	Diabetic foot	1,127 (0.2)
FC_024	Diseases of endocrine glands (including diabetes)	60,893 (8.8)
FC_025	Venous vascular disease	3,613 (0.5)
FC_032	Hereditary and degenerative diseases of the central nervous system	11,830 (1.7)
FC_034	Special bed provided by the Health Regional System	2,872 (0.4)
FC_037	Hypertension	156,560 (22.7)
FC_038	Ill-defined and unknown causes of morbidity and mortality	11,499 (1.7)
FC_043	Ischemic myocardial disease	30,939 (4.5)
FC_050	Chronic kidney disease	11,748 (1.7)
FC_051	Neurotic disorders, personality disorders, and other nonpsychotic mental disorders	8,682 (1.3)
FC_053	Nurse care provided at home	10,303 (1.5)
FC_057	Open wound of the lower limb	5,964 (0.9)
FC_058	Organic psychotic conditions	16,031 (2.3)
FC_062	Bacterial diseases	10,799 (1.6)
FC_064	Diseases of the urinary system	28,265 (4.1)
FC_066	Heart diseases	90,655 (13.2)
FC_069	Psychosis	9,514 (1.4)
FC_070	Diabetes supplies	10,263 (1.5)
FC_077	Pneumonia and influenza	49,539 (7.2)
FC_090	Transportation services including ambulance	2,152 (0.3)
FC_092	Walking aids and attachments	6,382 (0.9)
FC_093	Wheelchairs	3,776 (0.5)
FC_100	Osteoporosis (fragility fractures)	19,062 (2.8)
FC_114	Hearing impairment	4,727 (0.7)
FC_115	Visual impairment	1,100 (0.2)
FC_118	Cancer	48,641 (7.1)
FC_125	Dependency in self-care	12,738 (1.8)
FC_126	Dependency in self-dressing	12,657 (1.8)
FC_127	Dependency in walking	13,022 (1.9)
FC_128	Dependency in toileting	11,679 (1.7)
FC_129	Dependency in self feeding	9,193 (1.3)
FC_133	Living in a nursing home	22,050 (3.2)
FC_134	Living alone	376 (0.1)
FC_135	Low income	
	Upper-middle and high income	383,161 (55.6)
	Lower-middle income	189,939 (27.6)
	Low income	116,097 (16.8)
FC_137	End-stage renal disease (hemodialysis)	2,324 (0.3)

### Validation of RHD-FI versus c-FI and outcomes

**Figure 2:**
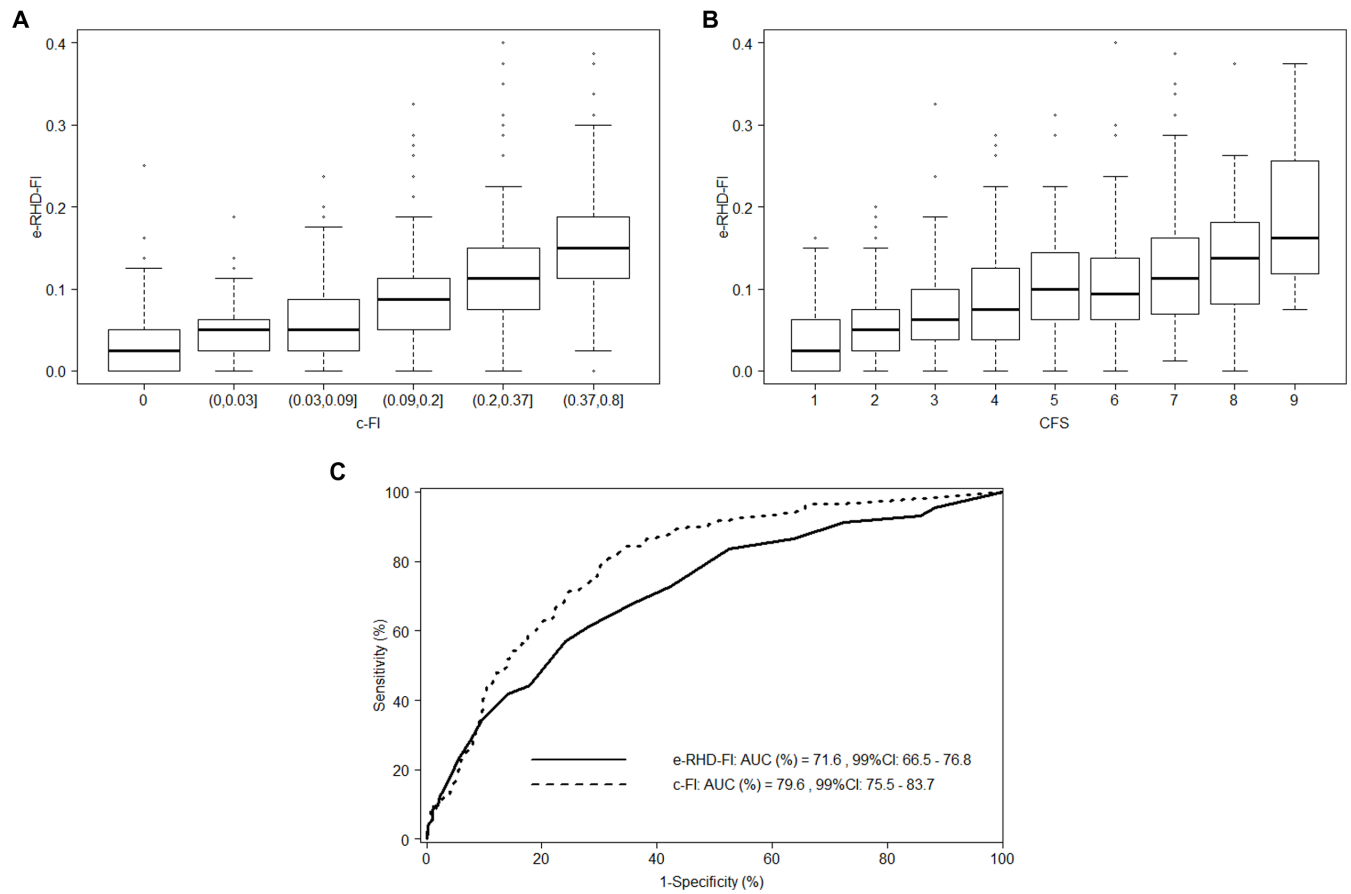
Distribution of the electronic-Regional Healthcare Database Frailty Index (e-RHD-FI) according to Clinical Frailty index (c-FI), Clinical Frailty Scale (CFS) and in-hospital mortality in the FRACOVID patients. Panel **A**: boxplot of e-RHD-FI according to the c-FI categorized using its 10th, 25th, 50th, 75th and 90th percentile values. Panel **B**: boxplot of e-RHD-FI according to the CFS levels. Panel **C**: ROC curve on in-hospital mortality according to e-RHD-FI (solid line) and c-FI (dotted line).

We matched the e-RHD and the clinical data in the cohort of 1,173 patients (median age 68 years, first-third quartile, 56–79, 63.2% males) recruited in the FRACOVID study, observing good agreement (close to 80%, see Supplementary Table S3) between the variables of e-RHD-FI and c-FI. The overall agreement for hypertension was 78%. The variable “visual loss” had an overall agreement of 95%: among the 1,091 patients who did not report visual loss in the FRACOVID study, almost all (1,090, 99.9%) had no visual loss according to the e-RHD. Conversely, among the 65 patients who reported visual impairment in the FRACOVID study, only 5 (7.7%) had visual impairment according to e-RHD. The median e-RHD-FI was 0.06 (first-third quartile, 0.04–0.11), compared to a median c-FI of 0.09 (first-third quartile, 0.03–0.20). The e-RHD-FI showed a strong correlation with the c-FI (Figure 2A, Spearman rho 0.59, 95%CI: 0.55–0.63) with a trend toward lower values for the e-RHD-FI. The e-RHD-FI was associated with the CFS score (Figure 2B, Spearman rho 0.44, 95%CI: 0.39–0.49). Figure 2C compares the performance of e-RHD-FI and c-FI in predicting in-hospital mortality. The AUC for e-RHD-FI was 71.6% (99%CI: 66.4–76.8%), lower than c-FI (AUC = 79.6, 99%CI: 75.5–83.7%, Figure 2C). e-RHD-FI was associated with in-hospital mortality after adjustment for potential confounders, with a HR of 1.39 (99%CI, 1.09–1.78, Supplementary Table S4) per 0.1-point increment of e-RHD-FI, similar to the FRACOVID study (HR = 1.29, 95%CI: 1.16–1.43).

### Association between e-RHD-FI and adverse health outcomes in adults with SARS-CoV-2

**Figure 3:**
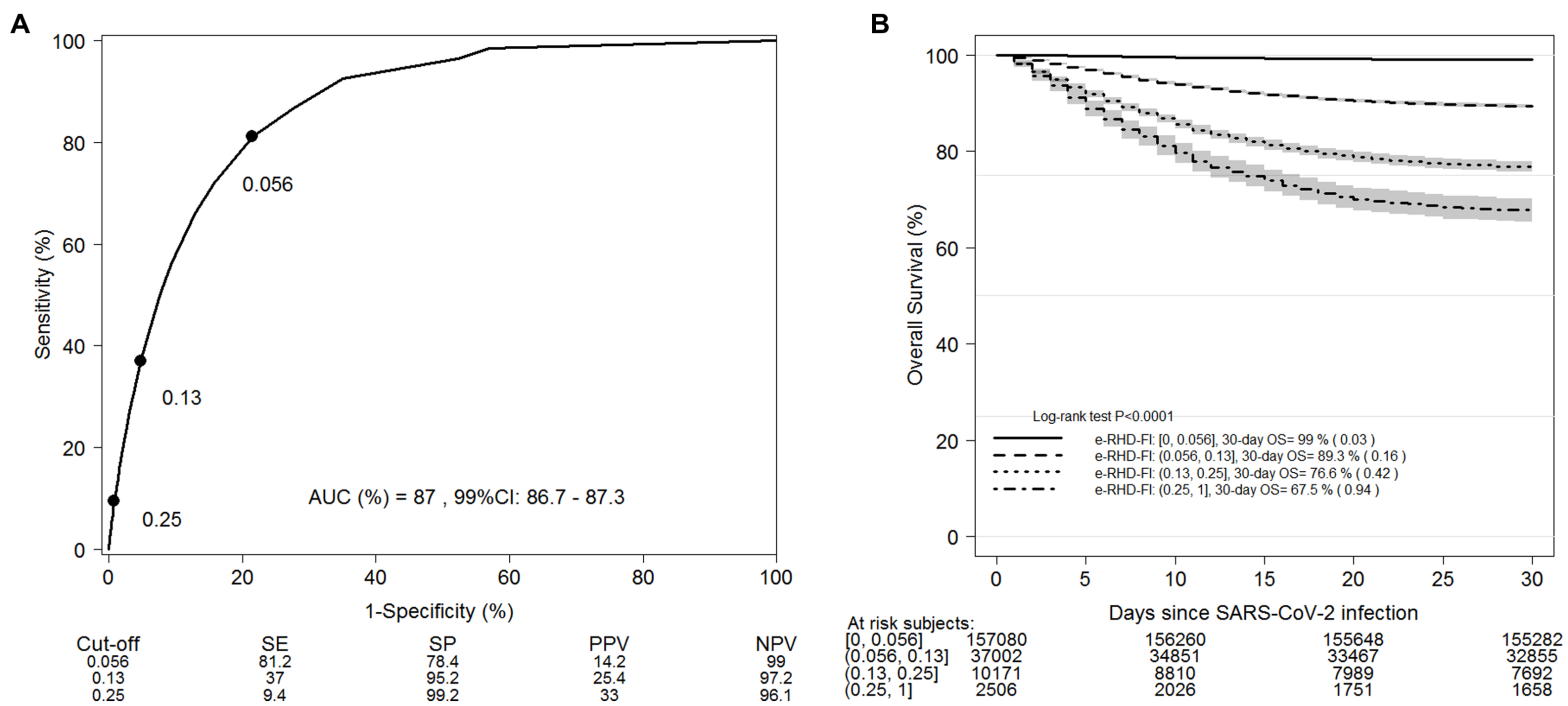
ROC curve for 30-day mortality according to e-RHD-FI on the training set (n=482,438) of adults infected by SARS-CoV-2 up to 20th May 2021 **(A)** and Kaplan-Meier survival estimate on the validation set (n=206,759) stratified by different proposed cut-offs of e-RHD-FI **(B)**. SE= Sensitivity; SP= Specificity; PPV: Positive Predictive Value; NPV: Negative Predictive Value.

482,438 adults, of whom 20,392 (4.2%) died within 30 days of a positive result at the nasopharyngeal swab test, were included in the training set. The AUC for 30-day mortality was 87% (99%CI: 86.7–87.3%) for e-RHD-FI and 57.9% (99%CI: 57.6–58.2%) for CCI. E-RHD-FI performed better than CCI in predicting mortality in adults younger than 50 years (84, 99%CI: 86.7–87.3% for e-RHD-FI versus 59.9, 99%CI: 56.2–63.6% for CCI). The optimal e-RHD-FI cut-off for mortality prediction was 0.056, with a sensitivity of 81.2% (99%CI 80.7–81.9%) and specificity of 78.5% (99%CI 78.3–78.6%). 206,759 adults, with 8,739 (4.2%) deaths within 30 days, were included in the validation set. When the cut-off was applied to stratify the validation set, the two strata showed significantly different mortality (log-rank *p* < 0.001). The ROC curve for the validation set is shown in Figure 3A, along with the predictive performance of three different cut-offs. The 30-day survival estimates for the four groups defined using these cut-offs are shown in Figure 3B. The groups with the lowest (≤0.056) and highest (>0.25) e-RHD-FI scores had 99% (99%CI: 98.9–99.1%, standard error (SE) = 0.03%) and 67.5% (99%CI: 65–69.9%, SE = 0.9%) 30-day survival, respectively. In a multivariable model adjusted for male sex, age, and period of diagnosis, the association between the e-RHD-FI score and 30-day mortality was significant (HR per 0.1-point increment of e-RHD-FI = 1.45, 99%CI: 1.42–1.47) (Table 2). The association between e-RHD-FI and 30-day mortality remained significant, even after stratifying for age class (Supplementary Table S5, hospital admission (Supplementary Table S6), and period of diagnosis (Supplementary Table S7). In the multivariable model, e-RHD-FI was also significantly associated with a higher risk of hospitalization (HR per 0.1-point increment = 1.47, 99%CI: 1.46–1.49), and with the WHO clinical progression scale. For each 0.1-point increment of the e-RHD-FI, there was a 1.84-fold increase in the odds of worsening by one point at the WHO Clinical Progression Scale (Table 2).

**Table 2:** Adjusted Cox regression models for 30-day mortality, 30-day hospitalization, the composite of both, and ordinal logistic regression for WHO clinical progression scale of 689,197 adults with SARS-CoV-2.

*N* = 689,197	30-day mortality (29,131)		30-day hospitalization (127,558)		Composite outcome (133,325)		WHO clinical progression scale^*^	
Parameter	HR(99%CI)	*p*	HR(99%CI)	*p*	HR(99%CI)	*p*	OR(99%CI)	*p*
eRHD-FI (per 0.1-point increment)	1.45 (1.42–1.47)	<0.001	1.47 (1.46–1.49)	<0.001	1.47 (1.45–1.48)	<0.001	1.84 (1.80–1.87)	<0.001
Male (Yes vs. No)	2.59 (2.51–2.68)	<0.001	1.57 (1.54–1.59)	<0.001	1.54 (1.52–1.57)	<0.001	1.85 (1.81–1.89)	<0.001
Age (years)	1.09 (1.09–1.09)	<0.001	1.04 (1.04–1.04)	<0.001	1.04 (1.04–1.04)	<0.001	1.02 (1.02–1.02)	<0.001
Diagnosis period (after 1st July 2020 vs. before)	0.38 (0.37–0.39)	<0.001	3.78 (3.68–3.89)	<0.001	2.99 (2.92–3.07)	<0.001	0.18 (0.17–0.18)	<0.001

^*^Scoring of the WHO clinical progression scale for COVID-19: non-hospitalization, hospitalization, admission to intensive care unit, death.

## Discussion

We developed and validated an e-RHD-FI, using information from the electronic healthcare database of the Lombardy region, Italy. The e-RHD-FI was developed according to the standardized criteria recommended by Searle et al. (16). The e-RHD-FI was validated against the c-FI and the CFS, using a set of clinical data prospectively collected in a multicentre study of patients hospitalized for COVID-19 (6). Overall, the study showed that e-RHD-FI had good performance in predicting in-hospital and 30-day mortality, risk of hospital admission, and worsening on the WHO clinical scale in COVID-19 patients.

Previous studies investigated the predictive power of the electronic FI (e-FI) for adverse outcomes in both hospitalized and community-dwelling individuals. Mak and colleagues developed a 48-item e-FI from routinely collected health records of 13,188 hospitalized older adults in Sweden, finding that a 0.03 increment in e-FI was independently associated with increased risk of in-hospital, 30-day, and 6-month mortality (35). Previously, Clegg and colleagues demonstrated that a 36-item e-FI was able to robustly predict mortality, hospitalization, and nursing home admission in a cohort of 931,541 older community-dwellers (24). Other studies, which computed an automated FI based on electronic health records, have been conducted in Canada (36), the United States (37), the United Kingdom (38), and France (39).

However, only one study used a database-derived e-FI (i.e., the Hospital Frailty Risk Score) to assess mortality in an English cohort of hospitalized older people affected by COVID-19 (40). To the best of our knowledge, this is therefore the first study to use an e-RHD-FI for predicting mortality in an adult community-dwelling population with SARS-CoV-2.

There are several findings from this study that should be highlighted. It is noteworthy that our e-RHD-FI had one of the highest performances in predicting mortality, compared to other electronic FIs. This result suggests that the variables included in our e-RHD-FI were adequately selected to represent the multiple dimensions of frailty. In fact, the e-RHD-FI included not only a list of chronic diseases usually considered in models predicting mortality but also several other conditions, known to increase susceptibility to adverse events (i.e., poor socio-economic status, sensory, cognitive, and functional impairments). Previous electronic FIs did not consider all these domains. For instance, four of the FIs included in a recent systematic review (41) did not include socioeconomic status (24, 38, 39, 42), which is a determinant of health status, whereas two other studies did not include cognitive problems or functional status (27, 37). Given its composition, we believe that our e-RHD-FI can also be proposed for non-COVID-19 patients, but future studies are required.

Another key finding of our study is that the AUC of the ROC curve for 30-day mortality was higher in the general population (AUC = 87%) than in adults hospitalized for COVID-19. In hospitalized patients, e-RHD-FI showed similar predictive power for short-term mortality to the FRACOVID study (6). The fact that frail elderly patients were less likely to be hospitalized during the pandemic, compared with robust counterparts, may partially explain our results. The e-RHD-FI was applied to the whole population, and not just to elderly patients, showing very good performance in younger adults. We also found that the ability of e-RHD-FI to predict 30-day mortality was higher than CCI, suggesting the potential benefits of assessing frailty in younger patients.

Other findings should be mentioned too. The agreement between each variable included in e-RHD-FI and c-FI was high. We found low sensitivity, but good or even excellent specificity for most of the variables included in the e-RHD-FI compared to clinical data. This was probably because electronic databases report health deficits only when they are highly pronounced. For example, in our e-RHD-FI, patients were classified as visually impaired only if they satisfied one of the two following criteria: (i) the patient benefited from an exemption (which is granted by the regional health system when contact lenses, eyeglasses, or Braille devices are required); ii) the patient had a diagnosis of visual impairment in medical records at hospital discharge. e-RHD-FI showed lower performance than c-FI in predicting in-hospital mortality (71.6% vs. 79.6%), likely because electronic databases are not as accurate as clinical databases in reporting health deficits.

The e-RHD-FI cut-offs used in our study had a low positive predictive value (14.2%) but a very high negative predictive value (99%), suggesting good performance in detecting individuals at low risk of death after a diagnosis of SARS-CoV-2. The 0.25 cut-off had optimal performance in identifying those at high risk of mortality (33%) and a low negative predictive value (96.1%), indicating that individuals with e-RHD-FI ≤ 0.25 had a 3.9% risk of 30-day mortality.

The results of this study may be useful, from a healthcare governance perspective, for managing frailty not only during but also after the pandemic. Currently, in the Italian National Health System, frail subjects are identified based only on generic characteristics, such as old age or the co-occurrence of chronic diseases. However, this approach is unable to capture the true nature of frailty and therefore is inadequate to target individuals who can truly benefit from specific interventions for frailty. The e-RHD-FI may serve this purpose, by enabling the Regional Health System to identify frail persons in a timely and simple manner. For instance, e-RHD-FI can be used to target people who will benefit most from vaccines. In this regard, surveillance systems based on predictive models adopted in restricted geographical areas have successfully prevented disease progression among people during the COVID-19 pandemic (1). The e-RHD-FI may also be used, on an individual basis, to identify individuals in need of coordinated and integrated interventions. On a regional level, the e-RHD-FI may help compare patient case mix and healthcare resources utilization across hospitals, according to frailty levels. This could have an important economic impact, given that frailty is associated with increased utilization of healthcare resources (10).

A strength of this study is that we used a large population-based healthcare database. Furthermore, we validated the e-RHD-FI against a FI based on *ad hoc* clinical data. We measured the accuracy of the deficits included in the e-RHD and the association between the e-RHD-FI and other well-known tools used to assess frailty (c-FI and CFS). This study also has some limitations. First, the e-RHD-FI was validated only in individuals with SARS-CoV-2 by May 20, 2021, thus excluding those who developed SARS-CoV-2 afterwards. We cannot exclude that the large fluctuations in COVID-19 infection and mortality rates observed during the pandemic, as well as the advent of vaccination, may have influenced our results. However, we expect the index to show good performance also in those who were positive for SARS-COV-2 during the third and fourth waves, as the index performance did not change significantly when we applied stratification for time of diagnosis. Furthermore, e-RHD-FI showed lower values than c-FI, possibly underestimating frailty. This might be due to the fact that it was not possible to retrieve complete information on all health deficits and determinants from administrative data (e.g., mental health). Moreover, we were unable to retrieve all the variables included in the c-FI (such as smoking status). However, the reduction in the prognostic performance was limited when comparison with c-FI was applied. Finally, the data refer only to the Lombardy region, therefore our results are not generalizable to the whole country.

To conclude, e-RHD-FI can predict mortality in community-dwelling adults with SARS-CoV-2 and can be used to target people at risk for future pandemics. Our findings also support the need to assess frailty with regional healthcare databases to implement specific approaches for frail people.

## Data availability statement

The datasets presented in this article are not readily available because they need an agreement to be accessed. Requests to access the datasets should be directed to Regione Lombardia.

## Ethics statement

The studies involving human participants were reviewed and approved by the Brianza Institutional Review Board under the number 3356-07/08/2020.

## Author contributions

PR, CS, GB, and MGV contributed to the conception and design of the study. AZ, EF, AM, PR, GO, MGV and GB contributed to the acquisition of the data. PR, GO, and MGV were responsible for the statistical analysis. PR, CS, FB, OL, FC, AZ, EF, AM, GB, and MGV were responsible for the interpretation of the data. PR, CS, AZ, EF, AM, GB, and MGV contributed to the draft of this paper. All authors have read, revised, and approved the final manuscript.

## Funding

This work was supported by a grant from the Cariplo (Cassa di Risparmio delle Province Lombarde) Foundation, Lombardia Region, Italy (The FRACOVID study, ClinicalTrials.gov Identifier: NCT04412265).

## Conflict of interest

The authors declare that the research was conducted in the absence of any commercial or financial relationships that could be construed as a potential conflict of interest.

## Publisher’s note

All claims expressed in this article are solely those of the authors and do not necessarily represent those of their affiliated organizations, or those of the publisher, the editors and the reviewers. Any product that may be evaluated in this article, or claim that may be made by its manufacturer, is not guaranteed or endorsed by the publisher.

## References

[1] Russo AG Faccini M Bergamaschi W Riussi A. Strategy to reduce adverse health outcomes in subjects highly vulnerable to COVID-19: results from a population-based study in northern Italy. BMJ Open. (2021) 11:e046044. doi: 10.1136/bmjopen-2020-046044, PMID: 33692188 PMC7948154

[2] Russo AG Decarli A Valsecchi MG. Strategy to identify priority groups for COVID-19 vaccination: a population based cohort study. Vaccine. (2021) 39:2517–25. doi: 10.1016/j.vaccine.2021.03.076, PMID: 33824037 PMC7997303

[3] Montero-Odasso M Hogan DB Lam R Madden K MacKnight C Molnar F. Age alone is not adequate to determine healthcare resource allocation during the COVID-19 pandemic. Can Geriatr J. (2020) 23:152–4. doi: 10.5770/cgj.23.452, PMID: 32550953 PMC7279701

[4] World Health Organization. Clinical consortium on healthy ageing: topic focus: frailty and intrinsic capacity. Geneva: World Health Organization (2016).

[5] Rutenberg AD Mitnitski AB Farrell SG Rockwood K. Unifying aging and frailty through complex dynamical networks. Exp Gerontol. (2018) 107:126–9. doi: 10.1016/j.exger.2017.08.027, PMID: 28847723

[6] Rebora P Focà E Salvatori A Zucchelli A Ceravolo I Ornago AM. The effect of frailty on in-hospital and medium-term mortality of patients with CoronaVIrus Disease-19: the FRACOVID study. Panminerva Med. (2022) 64. doi: 10.23736/S0031-0808.21.04506-7,On behalf of the FRACoViD Team 34761887

[7] Galimberti S Graziano F Maas AIR Isernia G Lecky F Jain S. Effect of frailty on 6-month outcome after traumatic brain injury: a multicentre cohort study with external validation. Lancet Neurol. (2022) 21:153–62. doi: 10.1016/S1474-4422(21)00374-4, PMID: 35065038

[8] Hanlon P Nicholl BI Jani BD Lee D McQueenie R Mair FS. Frailty and pre-frailty in middle-aged and older adults and its association with multimorbidity and mortality: a prospective analysis of 493 737 UK biobank participants. Lancet Public Health. (2018) 3:e323–32. doi: 10.1016/S2468-2667(18)30091-4, PMID: 29908859 PMC6028743

[9] Loecker C Schmaderer M Zimmerman L. Frailty in young and middle-aged adults: an integrative review. J Frailty Aging. (2021) 10:327–33. doi: 10.14283/jfa.2021.14, PMID: 34549246

[10] Dent E Martin FC Bergman H Woo J Romero-Ortuno R Walston JD. Management of frailty: opportunities, challenges, and future directions. Lancet. (2019) 394:1376–86. doi: 10.1016/S0140-6736(19)31785-4, PMID: 31609229

[11] SIMG (2021). Available at: https://www.simg.it/linea-guida-inter-societaria-per-la-gestione-della-multimorbilita-e-polifarmacoterapia-2021/

[12] Farmer C Fenu E O’Flynn N Guthrie B. Clinical assessment and management of multimorbidity: summary of NICE guidance. BMJ. (2016) 21:i4843. doi: 10.1136/bmj.i4843 27655884

[13] Ellis G Gardner M Tsiachristas A Langhorne P Burke O Harwood RH. Comprehensive geriatric assessment for older adults admitted to hospital. Cochrane Database Syst Rev. (2017) 9:CD006211. doi: 10.1002/14651858.CD006211.pub3 28898390 PMC6484374

[14] Rockwood K Song X MacKnight C Bergman H Hogan DB McDowell I. A global clinical measure of fitness and frailty in elderly people. CMAJ. (2005) 173:489–95. doi: 10.1503/cmaj.050051, PMID: 16129869 PMC1188185

[15] Mitnitski AB Mogilner AJ Rockwood K. Accumulation of deficits as a proxy measure of aging. Sci World J. (2001) 1:323–36. doi: 10.1100/tsw.2001.58, PMID: 12806071 PMC6084020

[16] Searle SD Mitnitski A Gahbauer EA Gill TM Rockwood K. A standard procedure for creating a frailty index. BMC Geriatr. (2008) 8:24. doi: 10.1186/1471-2318-8-24 18826625 PMC2573877

[17] Kundi H Çetin EHÖ Canpolat U Aras S Celik O Ata N. The role of frailty on adverse outcomes among older patients with COVID-19. J Infect. (2020) 81:944–51. doi: 10.1016/j.jinf.2020.09.029, PMID: 33002560 PMC7521439

[18] Aw D Woodrow L Ogliari G Harwood R. Association of frailty with mortality in older inpatients with Covid-19: a cohort study. Age Ageing. (2020) 49:915–22. doi: 10.1093/ageing/afaa184, PMID: 32778870 PMC7454254

[19] Brill SE Jarvis HC Ozcan E Burns TLP Warraich RA Amani LJ. COVID-19: a retrospective cohort study with focus on the over-80s and hospital-onset disease. BMC Med. (2020) 18:194. doi: 10.1186/s12916-020-01665-z, PMID: 32586323 PMC7315690

[20] Chinnadurai R Ogedengbe O Agarwal P Money-Coomes S Abdurrahman AZ Mohammed S. Older age and frailty are the chief predictors of mortality in COVID-19 patients admitted to an acute medical unit in a secondary care setting- a cohort study. BMC Geriatr. (2020) 20:409. doi: 10.1186/s12877-020-01803-5, PMID: 33066750 PMC7563906

[21] Hägg S Jylhävä J Wang Y Xu H Metzner C Annetorp M. Age, frailty, and comorbidity as prognostic factors for short-term outcomes in patients with coronavirus disease 2019 in geriatric care. J Am Med Dir Assoc. (2020) 21:1555–1559.e2. doi: 10.1016/j.jamda.2020.08.014, PMID: 32978065 PMC7427570

[22] Fernandes AL Pereira RMR. Frailty in the context of COVID-19 pandemic: a life-threatening condition. Front Med. (2022) 9:965562. doi: 10.3389/fmed.2022.965562 PMC9451136 36091682

[23] Kim DH. Measuring frailty in health care databases for clinical care and research. Ann Geriatr Med Res. (2020) 24:62–74. doi: 10.4235/agmr.20.0002, PMID: 32743326 PMC7370795

[24] Clegg A Bates C Young J Ryan R Nichols L Ann Teale E. Development and validation of an electronic frailty index using routine primary care electronic health record data. Age Ageing. (2016) 45:353–60. doi: 10.1093/ageing/afw039, PMID: 26944937 PMC4846793

[25] Regione Lombardia (2017). Riordino della rete di offerta e modalità di presa in carico dei pazienti cronici e/o fragili [Internet]. X/6551. Available at: https://www.regione.lombardia.it/wps/wcm/connect/e8579ec8-458b-4a81-975f-894dd2be9770/Delibera+n.+X_6551+del+04_05_2017.pdf?MOD=AJPERES&CACHEID=ROOTWORKSPACE-e8579ec8-458b-4a81-975f-894dd2be9770-m3lXaIa

[26] Regione Lombardia (2017). Governo della domanda: avvio della presa in carico di pazienti cronici e fragili [Internet]. Resolutions X/6164. Available at: https://www.regione.lombardia.it/wps/wcm/connect/f2ec5853-447c-4fc2-b4d4-e36d651ebbfb/delibera+6164_300117.pdf?MOD=AJPERES&CACHEID=ROOTWORKSPACE-f2ec5853-447c-4fc2-b4d4-e36d651ebbfb-m3lXCz3

[27] Bertini F Bergami G Montesi D Veronese G Marchesini G Pandolfi P. Predicting frailty condition in elderly using multidimensional socioclinical databases. Proc IEEE. (2018) 106:723–37. doi: 10.1109/JPROC.2018.2791463

[28] Silan M Caperna G Boccuzzo G. Link to external site this link will open in a new window. Quantifying frailty in older people at an Italian local health unit: a proposal based on partially ordered sets. Soc Indic Res. (2019) 146:757–82. doi: 10.1007/s11205-019-02142-8

[29] Rockwood K Mitnitski A. Frailty in relation to the accumulation of deficits. J Gerontol A Biol Sci Med Sci. (2007) 62:722–7. doi: 10.1093/gerona/62.7.722, PMID: 17634318

[30] Blakely T Salmond C. Probabilistic record linkage and a method to calculate the positive predictive value. Int J Epidemiol. (2002) 31:1246–52. doi: 10.1093/ije/31.6.1246, PMID: 12540730

[31] Sayers A Ben-Shlomo Y Blom AW Steele F. Probabilistic record linkage. Int J Epidemiol. (2016) 45:954–64. doi: 10.1093/ije/dyv322, PMID: 26686842 PMC5005943

[32] Song X Mitnitski A Rockwood K. Prevalence and 10-year outcomes of frailty in older adults in relation to deficit accumulation: frailty prevalence and outcome. J Am Geriatr Soc. (2010) 58:681–7. doi: 10.1111/j.1532-5415.2010.02764.x 20345864

[33] Perego S Zambon A Nistri S Bruni A Motta S Cavalieri D’Oro L. Prevalence, clinical correlates, and burden of undiagnosed aortic stenosis in older patients: a prospective study in a non-cardiologic acute hospital ward. Aging Clin Exp Res. (2020) 32:1533–40. doi: 10.1007/s40520-020-01471-w, PMID: 31970672

[34] Deyo R. Adapting a clinical comorbidity index for use with ICD-9-CM administrative databases. J Clin Epidemiol. (1992) 45:613–9. doi: 10.1016/0895-4356(92)90133-8, PMID: 1607900

[35] Mak JKL Hägg S Eriksdotter M Annetorp M Kuja-Halkola R Kananen L. Development of an electronic frailty index for hospitalized older adults in Sweden. J Gerontol A Biol Sci Med Sci. (2022) 77:2311–9. doi: 10.1093/gerona/glac069, PMID: 35303746 PMC9678204

[36] McIsaac DI Wong CA Huang A Moloo H van Walraven C. Derivation and validation of a generalizable preoperative frailty index using population-based health administrative data. Ann Surg. (2019) 270:102–8. doi: 10.1097/SLA.0000000000002769, PMID: 29672410

[37] Kim DH Glynn RJ Avorn J Lipsitz LA Rockwood K Pawar A. Validation of a claims-based frailty index against physical performance and adverse health outcomes in the health and retirement study. J Gerontol A Biol Sci Med Sci. (2019) 74:1271–6. doi: 10.1093/gerona/gly197, PMID: 30165612 PMC6625579

[38] Gilbert T Neuburger J Kraindler J Keeble E Smith P Ariti C. Development and validation of a hospital frailty risk score focusing on older people in acute care settings using electronic hospital records: an observational study. Lancet Lond Engl. (2018) 391:1775–82. doi: 10.1016/S0140-6736(18)30668-8, PMID: 29706364 PMC5946808

[39] Gilbert T Cordier Q Polazzi S Bonnefoy M Keeble E Street A. External validation of the hospital frailty risk score in France. Age Ageing. (2022) 51. doi: 10.1093/ageing/afab126 PMC8753041 34185827

[40] Maynou L Owen R Konstant-Hambling R Imam T Arkill S Bertfield D. The association between frailty risk and COVID-19-associated all-mortality in hospitalised older people: a national cohort study. Eur Geriatr Med. (2022) 13:1149–57. doi: 10.1007/s41999-022-00668-8 35750959 PMC9244480

[41] Nghiem S Sajeewani D Henderson K Afoakwah C Byrnes J Moyle W. Development of frailty measurement tools using administrative health data: a systematic review. Arch Gerontol Geriatr. (2020) 89:104102. doi: 10.1016/j.archger.2020.104102 32464423

[42] Soong J Poots A Scott S Donald K Woodcock T Lovett D. Quantifying the prevalence of frailty in English hospitals. BMJ Open. (2015) 5:e008456. doi: 10.1136/bmjopen-2015-008456, PMID: 26490097 PMC4621378

