# Supplementary material for: Development and validation of an electronic database-based frailty index to predict mortality and hospitalization in a population-based study of adults with SARS-CoV-2
Source: Front Med (Lausanne). 2023 May 12;10:1134377. doi: 10.3389/fmed.2023.1134377 (PMC10213394; doi:10.3389/fmed.2023.1134377)
Supplement: Supplementary file 1 [file Data_Sheet_1.docx]

Supplementary material

# Frailty index derivation on Regional Healthcare Databases

The process of index derivation followed a multistep strategy as summarized in Figure S1 below.

Source mapping

identification of data sources

classification systems

Identification of deficits

List of candidate deficits

Measurability in RHD

Algorithm development

Existing algorithms

*ad hoc* algorithms

*Figure S1:* Process of index derivation

## Source Mapping

Based on the availability of the RHDs, the data sources served to gather relevant information about health deficits as described in Supplementary Table S1.

*Table S1: Data sources and classification used*

| Source | Information | Classification |
| --- | --- | --- |
| Demographics | Age, gender, vital status | N/A |
| Hospital discharge form (HDF) | Diagnoses | ICD9-CM |
|  | Procedures | ICD9-CM |
| Outpatient services | Procedures | ICD9-CM (extended) |
| Emergency departments | Diagnoses | ICD9-CM |
| Exemption database | Certification of chronic diseases | ICD9-CM |
|  | Certification of disability | 1-100% |
|  | Income level | €/year |
| Pharmacy | Drug delivery (outpatient) | ATC |
| Prosthetics and orthotics | Delivery of prosthetics and orthotics | EN ISO 9999 |
| Intermediate Assistance Observation Sheet | Nursing Home admission | N/A |
|  | Diagnoses | ICD9-CM |
| Integrated Home Assistance | Home nursing care service | N/A |
|  | Diagnoses | ICD9-CM |
|  | Activities of Daily Living | 1-3 levels of dependency |
| Individual disabled sheet | Diagnoses | ICD9-CM |
| Psychiatric services | Diagnoses | ICD9-CM |
| Health Emergency System | Transportation services | N/A |

N/A, not applicable; ICD9-CM, International Classification of Diseases, 9^th^ Revision, Clinical Modification; ATC, Anatomical Therapeutic Chemical classification; EN ISO 9999, classification and terminology of assistive products.

## Identification of deficits

Three different sources contributed to the list of candidate deficits to be included in the RHD- based frailty index (FI)

- The 36-item FI developed in the context of the FRACOVID study [(on behalf of the FRACoViD Team et al. 2022)](https://www.zotero.org/google-docs/?7jPynG)
- The 27-item deficits from the claim-based FI by Kim et al. (2018)[(Kim et al. 2018)](https://www.zotero.org/google-docs/?k4Rk7U)
- A socio-economic status indicator.

After removing duplicates and evaluating the potential measurability of the individual deficits by administrative data, a final list of 40 deficits was defined.

## Algorithm development

For each item, an algorithm was defined in different ways:

- Adaptation of the Kim algorithms to the Lombardy RHD sources[(Kim et al. 2018)](https://www.zotero.org/google-docs/?Z9qLes)
- Application of the regional healthcare database algorithms to identify chronic conditions (Resolution X/6164 of 30 January 2017 “Governo della domanda: avvio della presa in carico di pazienti cronici e fragili” and X/6551 of 4 May 2017 “Riordino della rete di offerta e modalità di presa in carico dei pazienti cronici e/o fragili”)
- Ad hoc development of algorithms to identify deficits.

As a general rule, to identify deficits associated with chronic disease conditions, we applied the following recall periods from the index date: 10 years for chronic disease certifications (e.g. arterial hypertension); 5 years for chronic conditions diagnosed during inpatient and outpatient encounters (e.g. chronic obstructive pulmonary disease); 1 year for drug and prosthetic delivery-derived indicators and for acute events associated with the concept of frailty (e.g. open wound of the lower limb).

The details of the algorithms are reported in Table S2.

# References

[Kim, Dae Hyun, Sebastian Schneeweiss, Robert J. Glynn, Lewis A. Lipsitz, Kenneth Rockwood, and Jerry Avorn. 2018. “Measuring Frailty in Medicare Data: Development and Validation of a Claims-Based Frailty Index.” *The Journals of Gerontology Series A: Biological Sciences and Medical Sciences* 73(7):980–87. doi: 10.1093/gerona/glx229.](https://www.zotero.org/google-docs/?QOJq0s)

[on behalf of the FRACoViD Team, Paola Rebora, Emanuele Focà, Andrea Salvatori, Alberto Zucchelli, Isabella Ceravolo, Alice M. Ornago, Alberto Finazzi, Stefania Arsuffi, Paolo Bonfanti, Giuseppe Citerio, Paolo Mazzola, Fiona Ecarnot, Maria G. Valsecchi, Alessandra Marengoni, and Giuseppe Bellelli. 2022. “The Effect of Frailty on In-Hospital and Medium-Term Mortality of Patients with COronaVIrus Disease-19: The FRACOVID Study.” *Panminerva Medica* 64(1). doi: 10.23736/S0031-0808.21.04506-7.](https://www.zotero.org/google-docs/?QOJq0s)

Table S2

| Deficit ID | Description | Source | Classification system | Codes | recall period |
| --- | --- | --- | --- | --- | --- |
| FC_001 | Respiratory failure /Oxygen therapy | Exemption database | ICD-9-CM (modified) | 024.518.8;024.518.81 | 0-10 yrs |
|  |  | Pharmacy | ATC | V03AN01 | 0-1 yr |
|  |  | Prosthetics and orthotics | EN ISO 9999 | 030318000; (040318003; 040318006) | 0-1 yr |
| FC_009 | Arthritis and related disorders | Exemption database | ICD-9-CM (modified) | 006*; 028*; 030*; 045*; 054*; 067*; [RM0010; RM0020; RM0021; RM0030; RM0060; RM0120; RC0220; RC0110; RC0210; RG0020; RG0030; RG0050; RG0070; RG0080; RG0090] | 0-10 yrs |
|  |  | Emergency departments | ICD-9-CM | 710*-719* | 0-5 yrs |
|  |  | Hospital discharge form (HDF) | ICD-9-CM | 710*-719* | 0-5 yrs |
| FC_012 | Cerebrovascular disease | Exemption database | ICD-9-CM (modified) | 002.433;002.434;002.437 | 0-10 yrs |
|  |  | Emergency departments | ICD-9-CM | 430*-438* | 0-5 yrs |
|  |  | Hospital discharge form (HDF) | ICD-9-CM | 430*-438* | 0-5 yrs |
| FC_014 | Chronic obstructive pulmonary disease and allied conditions | Exemption database | ICD-9-CM (modified) | 007; 057; | 0-10 yrs |
|  |  | Pharmacy | ATC | R03* (DDD>30%) | 0-1 yr |
|  |  | Emergency departments | ICD-9-CM | 490*-496* | 0-5 yrs |
|  |  | Hospital discharge form (HDF) | ICD-9-CM | 490*-496* | 0-5 yrs |
| FC_017 | Contusion with intact skin surface | Emergency departments | ICD-9-CM | 920*-924* | 0-5 yrs |
|  |  | Hospital discharge form (HDF) | ICD-9-CM | 920*-924* | 0-5 yrs |
| FC_019 | Diabetic foot | Exemption database | ICD-9-CM (modified) | 013.250 | 0-10 yrs |
|  |  | Prosthetics and orthotics | EN ISO 9999 | exemption 013.250 AND 063303003-063307009 | 0-1 yr |
|  |  | Hospital discharge form (HDF) | ICD-9-CM | exemption 013.250 AND 84.10–84.19; 39.25, 39.29; 39.50, 39.90 | 0-5 yrs |
|  |  | Hospital discharge form (HDF) | ICD-9-CM | exemption 013.250 AND 440.23, 707.14, 707.15; 713.0, 713.5, 713.8; 6811, 6819, 6826, 6827, 6829, 730.07, 730.17, 730.27, 99.21; 440.20, 440.21, 440.22, 440.23, 440.29, 443.9, 785.4, 440.0, 440.24; | 0-5 yrs |
| FC_024 | Diseases of endocrine glands (including diabetes) | Exemption database | ICD-9-CM (modified) | 001*; 012*; 013*; 026*; 032*; 039*; 066* | 0-10 yrs |
|  |  | Emergency departments | ICD-9-CM | 250*-259* | 0-5 yrs |
|  |  | Hospital discharge form (HDF) | ICD-9-CM | 250*-259* | 0-5 yrs |
| FC_025 | Venous vascular disease | Exemption database | ICD-9-CM (modified) | 002.452;002.453;002.459.1 | 0-10 yrs |
|  |  | Emergency departments | ICD-9-CM | 452*;453*;459.1* | 0-5 yrs |
|  |  | Hospital discharge form (HDF) | ICD-9-CM | 452*;453*;459.1* | 0-5 yrs |
| FC_032 | Hereditary and degenerative diseases of the central nervous system | Integrated Home Assistance | ICD-9-CM | 330*-337* | 0-5 yrs |
|  |  | Exemption database | ICD-9-CM (modified) | 029*; 038* | 0-10 yrs |
|  |  | Emergency departments | ICD-9-CM | 330*-337* | 0-5 yrs |
|  |  | Hospital discharge form (HDF) | ICD-9-CM | 330*-337* | 0-5 yrs |
|  |  | Individual disabled sheet | ICD-9-CM | 330*-337* | 0-5 yrs |
|  |  | Intermediate Assistance Observation Sheet | ICD-9-CM | 330*-337* | 0-5 yrs |
| FC_034 | Hospital beds | Prosthetics and orthotics | EN ISO 9999 | 181207003; 181207006; 181210003; 181210006; 181210009; 181212103; 181212106; 181227003; 181227103; 181291003; 181291006; 033306003; 033306006; 033306009; 033306012; 033306015; 033306018; 033306021; 043306006; 043306009; 043306012; 043306015; 043306018; 043306021 | 0-1 yr |
| FC_037 | Hypertension | Exemption database | ICD-9-CM (modified) | 031.401;031.405;D31.401;D31.405 | 0-10 yrs |
|  |  | Pharmacy | ATC | C02AC01;C02CA04;C03*;C07*;C08C*;C09* - (DDD > 50%) | 0-1 yr |
|  |  | Hospital discharge form (HDF) | ICD-9-CM | 401*;403*;405* | 0-5 yrs |
|  |  | Hospital discharge form (HDF) | ICD-9-CM | 134 | 0-5 yrs |
| FC_038 | Ill-defined and unknown causes of morbidity and mortality | Emergency departments | ICD-9-CM | 797*-799* | 0-5 yrs |
|  |  | Hospital discharge form (HDF) | ICD-9-CM | 797*-799* | 0-5 yrs |
| FC_043 | Ischemic myocardial disease | Exemption database | ICD-9-CM (modified) | 002.414 | 0-10 yrs |
|  |  | Pharmacy | ATC | C01DA*(DDD>50%) | 0-1 yr |
|  |  | Emergency departments | ICD-9-CM | 410*-414* | 0-5 yrs |
|  |  | Hospital discharge form (HDF) | ICD-9-CM | 410*-414* | 0-5 yrs |
|  |  | Hospital discharge form (HDF) | ICD-9-CM | 36* | 0-5 yrs |
| FC_050 | Chronic kidney disease | Exemption database | ICD-9-CM (modified) | 023*;031.403;031.404 | 0-10 yrs |
|  |  | Pharmacy | ATC | V03AE02;V03AE03;V03AE01;A11CC04;H05BX01;H05BX02 (DDD>50%) | 0-1 yr |
|  |  | Hospital discharge form (HDF) | ICD-9-CM | V56*;585*;586* | 0-5 yrs |
|  |  | Hospital discharge form (HDF) | ICD-9-CM | 316;317 | 0-5 yrs |
| FC_051 | Neurotic Disorders, Personality Disorders, And Other Nonpsychotic Mental Disorders | Integrated Home Assistance | ICD-9-CM | 300*-316* | 0-5 yrs |
|  |  | Psychiatric services | ICD-9-CM | 300*-316* | 0-5 yrs |
|  |  | Hospital discharge form (HDF) | ICD-9-CM | 300*-316* | 0-5 yrs |
|  |  | Individual disabled sheet | ICD-9-CM | 300*-316* | 0-5 yrs |
|  |  | Intermediate Assistance Observation Sheet | ICD-9-CM | 300*-316* | 0-5 yrs |
| FC_053 | Home nursing care | Integrated Home Assistance | encounter |  | 0-1 yr |
| FC_057 | Open wound of lower limb | Integrated Home Assistance | encounter |  | 0-1 yr |
|  |  | Integrated Home Assistance | ICD-9-CM | 890-897 | 0-1 yr |
|  |  | Outpatient services | ICD-9-CM (extended) | 96591; 96592; 96593; 96594; 96595; 96596 | 0-1 yr |
|  |  | Emergency departments | ICD-9-CM | 890-897 | 0-1 yr |
|  |  | Hospital discharge form (HDF) | ICD-9-CM | 890-897 | 0-1 yr |
| FC_058 | Organic psychotic conditions | Integrated Home Assistance | ICD-9-CM | 290-294 | 0-5 yrs |
|  |  | Exemption database | ICD-9-CM (modified) | 011* | 0-10 yrs |
|  |  | Psychiatric services | ICD-9-CM | 290-294 | 0-5 yrs |
|  |  | Hospital discharge form (HDF) | ICD-9-CM | 290-294 | 0-5 yrs |
|  |  | Individual disabled sheet | ICD-9-CM | 290-294 | 0-5 yrs |
|  |  | Intermediate Assistance Observation Sheet | ICD-9-CM | 290-294 | 0-5 yrs |
| FC_062 | Bacterial diseases | Emergency departments | ICD-9-CM | 030-041 | 0-5 yrs |
|  |  | Hospital discharge form (HDF) | ICD-9-CM | 030-041 | 0-5 yrs |
| FC_064 | Diseases of urinary system | Emergency departments | ICD-9-CM | 590-599 | 0-5 yrs |
|  |  | Hospital discharge form (HDF) | ICD-9-CM | 590-599 | 0-5 yrs |
| FC_066 | Heart diseases | Exemption database | ICD-9-CM (modified) | 002.424; 002.426; 002.427; 002.429.4; 021.428 | 0-10 yrs |
|  |  | Pharmacy | ATC | C09A*;C09C* (DDD>50%) | 0-1 yr |
|  |  | Pharmacy | ATC | C03CA*;C03CB*;C03EB* (DDD>50%) | 0-1 yr |
|  |  | Pharmacy | ATC | C07AG02;C07AB02;C07AB07 (DDD>50%) | 0-1 yr |
|  |  | Emergency departments | ICD-9-CM | 420-429 | 0-5 yrs |
|  |  | Hospital discharge form (HDF) | ICD-9-CM | 420-429 | 0-5 yrs |
| FC_069 | Psychoses | Integrated Home Assistance | ICD-9-CM | 295*-299* | 0-5 yrs |
|  |  | Exemption database | ICD-9-CM (modified) | 044.295.0; .295.1; 044.295.2; 044.295.3; 044.295.5; 044.295.6; 044.295.7; 044.295.8; 044.296.0; 044.296.1; 044.296.2; 044.296.3; 044.296.4; 044.296.5; 044.296.6; 044.296.7; 044.296.8; 044.297.0; 044.297.1; 044.297.2; 044.297.3; 044.297.8; 044.298.0; 044.298.1; 044.298.2; 044.298.4; 044.298.8; 044.299.0; 044.299.1; 044.299.8 | 0-10 yrs |
|  |  | Psychiatric services | ICD-9-CM | 295*-299* | 0-5 yrs |
|  |  | Hospital discharge form (HDF) | ICD-9-CM | 295*-299* | 0-5 yrs |
|  |  | Individual disabled sheet | ICD-9-CM | 295*-299* | 0-5 yrs |
|  |  | Intermediate Assistance Observation Sheet | ICD-9-CM | 295*-299* | 0-5 yrs |
| FC_070 | Diabetes supplies | Outpatient services (for diabetes) | encounter |  | 0-1 yrs |
| FC_077 | Pneumonia and influenza | Emergency departments | ICD-9-CM | 480*-487* | 0-5 yrs |
|  |  | Hospital discharge form (HDF) | ICD-9-CM | 480*-487* | 0-5 yrs |
| FC_090 | Transportation services including ambulance | Health Emergency System | Encounter |  | 0-1 yr |
| FC_092 | Walking aids and attachments | Prosthetics and orthotics | EN ISO 9999 | 120306003-121891033 | 0-5 yrs |
| FC_093 | Wheelchairs | Prosthetics and orthotics | EN ISO 9999 | 12.22*; 12.23*; 12.24*; 12.36* | 0-1 yr |
| FC_100 | Osteoporosis (fragility fractures) | Emergency departments | ICD-9-CM | 733.1*; 820*-821*; 805*; 813*-814* | 0-5 yrs |
|  |  | Hospital discharge form (HDF) | ICD-9-CM | 733.1*; 820*-821*; 805*; 813*-814* | 0-5 yrs |
| FC_114 | Hearing impairment | Exemption database | ICD-9-CM (modified) | C06 | 0-10 yrs |
|  |  | Prosthetics and orthotics | EN ISO 9999 | 2145* | 0-5 yrs |
|  |  | Hospital discharge form (HDF) | ICD-9-CM | 389* | 0-5 yrs |
| FC_115 | Visual impairment | Exemption database | ICD-9-CM (modified) | C05 | 0-10 yrs |
|  |  | Prosthetics and orthotics | EN ISO 9999 | 210303003-211515006 | 0-5 yrs |
|  |  | Hospital discharge form (HDF) | ICD-9-CM | 369* | 0-5 yrs |
| FC_118 | Cancer | Exemption database | ICD-9-CM (modified) | RB* | 0-5 yrs |
|  |  | Exemption database | ICD-9-CM (modified) | 048* | 0-5 yrs |
| FC_125 | Dependency in self-care | Integrated Home Assistance | 1-3 levels of dependency |  | 0-5 yrs |
| FC_126 | Dependency in self-dressing | Integrated Home Assistance | 1-3 levels of dependency |  | 0-5 yrs |
| FC_127 | Dependency in walking | Integrated Home Assistance | 1-3 levels of dependency |  | 0-5 yrs |
| FC_128 | Dependency in using toilette | Integrated Home Assistance | 1-3 levels of dependency |  | 0-5 yrs |
| FC_129 | Dependency in self feeding | Integrated Home Assistance | 1-3 levels of dependency |  | 0-5 yrs |
| FC_133 | Living in nursing home | Intermediate Assistance Observation Sheet | encounter |  | 0-1 yr |
| FC_134 | Living alone | Integrated Home Assistance | N/A |  | 0-5 yrs |
| FC_135 | Low income | Exemption database | N/A | Lower-middle income: E01, E05, E30, E40 contribute for 0.5  Low income: E02-E04, E12-E15 contribute for 1 | 0-1 yr |
| FC_137 | End stage renal disease (hemodialysis) | Outpatient services | ICD-9-CM (extended) | 3895; 39951; 39952; 39953; 39954; 39959; 54981; 54982; 89031; 3995A | 0-1 yr |

**Figure S2.** Flow-chart of patient selection


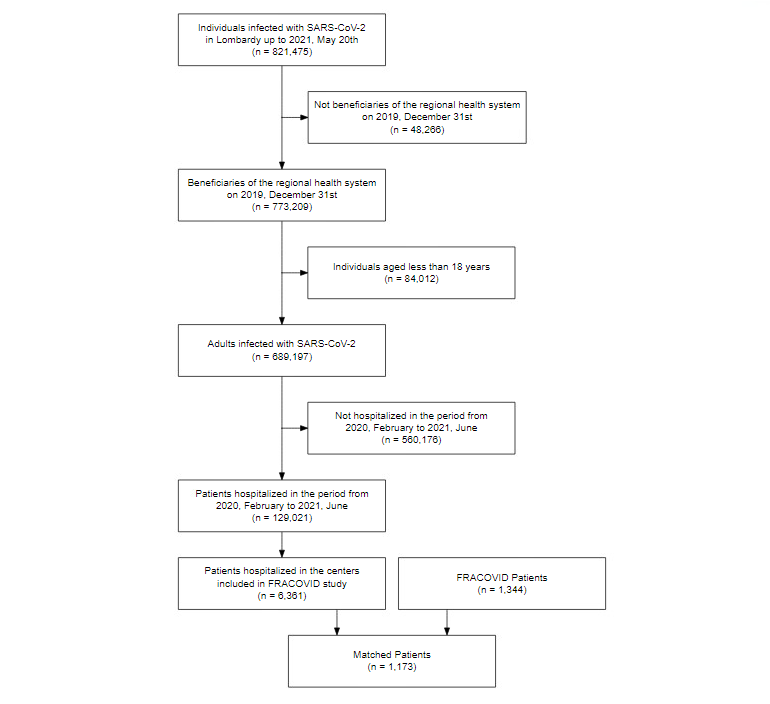


**Table S3.** Agreement between the information available in the RHD with information collected *ad hoc* regarding single deficits. Sensitivity and specificity are defined here as the percentage of adults with and without the deficit in the RHD among all adults declared with vs without the deficit, respectively, in the clinical data collected *ad hoc*.

| **Deficit ID** | **Description** | **N** | **Agreement, n (%)** | **Specificity (%)** | **Sensitivity (%)** |
| --- | --- | --- | --- | --- | --- |
| FC_014 | Chronic obstructive pulmonary disease and allied conditions | 1172 | 1051 (89.7) | 96.1 | 38.0 |
| FC_037 | Hypertension | 1172 | 917 (78.2) | 77.7 | 78.8 |
| FC_043 | Ischemic myocardial disease | 1170 | 963 (82.3) | 97.0 | 35.9 |
| FC_050 | Chronic kidney disease | 1171 | 1078 (92.1) | 97.8 | 31.7 |
| FC_092 | Walking aids and attachments | 1003 | 892 (88.9) | 91.1 | 11.0 |
| FC_093 | Wheelchairs | 1001 | 832 (83.1) | 99.5 | 2.9 |
| FC_100 | Osteoporosis (fracture) | 1158 | 1075 (92.8) | 97.4 | 14.3 |
| FC_114 | Hearing impairment | 1169 | 1090 (93.2) | 99.2 | 9.1 |
| FC_115 | Visual loss | 1156 | 1095 (94.7) | 99.9 | 7.7 |
| FC_118 | Cancer | 1171 | 1017 (86.8) | 91.5 | 56.2 |
| FC_133 | Living in a nursing home | 1025 | 946 (92.3) | 99.1 | 25.3* |

*The variable in the clinical data included also the presence of formal caregivers

# Probabilistic Matching

## A probabilistic model was defined using information on sex, age at infection and hospital admission (dates of admission and discharge, mortality and center) to link patients included in the FRACOVID study with their data in the RHD [(Blakely and Salmond 2002; Sayers et al. 2016)](https://www.zotero.org/google-docs/?9AQktT). Due to the large number of possible pairs, we considered as possible matched pairs only pairs with same sex, admitted to the same hospital and with coherent data on in-hospital mortality. A maximum difference of 1 year in age and of 7 days in the dates of admission and discharge was allowed before selecting the matches. We fixed at 0.95 the probability that true matches agreed for each linking variable (“m-probability”) and estimated the probability that false matches randomly agreed on each linking variable (“u-probability”) according to Fellegi and Sunter’s approach. Using these two probabilities, we calculated the complex agreement weight [(Sayers et al. 2016)](https://www.zotero.org/google-docs/?FUne1t) for each pair. Finally, we selected as matches only pairs with an agreement weight higher than the threshold (8.68) defined according to Cook et al. [(Cook et al. 2021)](https://www.zotero.org/google-docs/?39kbq1). If multiple regional records matched with a single FRACOVID patient, the match with the highest weight was selected.

[Blakely, Tony, and Clare Salmond. 2002. “Probabilistic Record Linkage and a Method to Calculate the Positive Predictive Value.” *International Journal of Epidemiology* 31(6):1246–52. doi: 10.1093/ije/31.6.1246.](https://www.zotero.org/google-docs/?QOJq0s)

[Cook, Michael J., Suzanne M. M. Verstappen, Mark Lunt, and Terence W. O’Neill. 2021. “Increased Frailty in People with Osteoarthritis and Rheumatoid Arthritis and the Influence of Co-Morbidity: An Analysis of the UK Biobank Cohort.” *Arthritis Care & Research*. doi: 10.1002/acr.24747.](https://www.zotero.org/google-docs/?QOJq0s)

[Sayers, Adrian, Yoav Ben-Shlomo, Ashley W. Blom, and Fiona Steele. 2016. “Probabilistic Record Linkage.” *International Journal of Epidemiology* 45(3):954–64. doi: 10.1093/ije/dyv322.](https://www.zotero.org/google-docs/?QOJq0s)

**Table** **S4.** Adjusted Cox regression model for in-hospital mortality in patients included in the FRACOVID study (matched set, N=1,173, number of in-hospital deaths = 206)

| **N=1,173** | **In-hospital mortality (N=206)** | |
| --- | --- | --- |
| **Parameter** | **HR (99%CI)** | **p-value** |
| RHD-FI (per 0.1-point increment) | 1.39 (1.09 – 1.78) | 0.0006 |
| Male (Yes vs No) | 1.71 (1.16 – 2.53) | 0.0004 |
| Age (years) | 1.08 (1.06 – 1.11) | <0.0001 |
| Admission period (after 1st July 2020 vs before) | 0.52 (0.36 – 0.76) | <0.0001 |

**Table** **S5.** Adjusted Cox regression models for 30-day mortality in 689,197 adults infected with SARS-CoV-2, stratified by age category

|  | **Mortality within 30 days** | | | | | |
| --- | --- | --- | --- | --- | --- | --- |
| **N=689,197**  **(events=29,131)** | **Age < 70 years**  **(n=542,485, events=3,382)** | | **Age 70 – 80 years**  **(n=75,347, events=8,534)** | | **Age > 80 years**  **(n=71,365, events=17,215)** | |
| **Parameter** | **HR**  **(99%CI)** | **p-value** | **HR**  **(99%CI)** | **p-value** | **HR**  **(99%CI)** | **p-value** |
| FI (per 0.1-point increment) | 2.41  (2.30 – 2.52) | <0.001 | 1.53  (1.48 – 1.58) | <0.001 | 1.26  (1.23 – 1.29) | <0.001 |
| Male (Yes vs No) | 2.24  (2.02 – 2.48) | <0.001 | 2.07  (1.95 – 2.20) | <0.001 | 2.21  (2.13 – 2.31) | <0.001 |
| Age (years) | 1.13  (1.12 – 1.14) | <0.001 | 1.08  (1.07 – 1.09) | <0.001 | 1.04  (1.04 – 1.05) | <0.001 |
| Diagnosis period (after 1st July 2020 vs before) | 0.14  (0.13 – 0.15) | <0.001 | 0.24  (0.23 – 0.26) | <0.001 | 0.57  (0.54 – 0.59) | <0.001 |

**Table** **S6.** Adjusted Cox regression models for 30-day mortality in 689,197 adults infected with SARS-CoV-2 stratified by hospitalization

|  | **Mortality within 30 days** | | | |
| --- | --- | --- | --- | --- |
| **N=689,197**  **(events=29,131)** | **Hospitalized**  **(n=129,021, events=23,366)** | | **Not hospitalized**  **(n=560,176, events=5,765)** | |
| **Parameter** | **HR**  **(99%CI)** | **p-value** | **HR**  **(99%CI)** | **p-value** |
| FI (per 0.1-point increment) | 1.27  (1.24 – 1.29) | <0.001 | 1.49  (1.44 – 1.55) | <0.001 |
| Male (Yes vs No) | 1.75  (1.69 – 1.81) | <0.001 | 2.23  (2.07 – 2.40) | <0.001 |
| Age (years) | 1.08  (1.07 – 1.08) | <0.001 | 1.13  (1.12 – 1.13) | <0.001 |
| Diagnosis period (after 1st July 2020 vs before) | 0.50  (0.48 – 0.51) | <0.001 | 0.39  (0.36 – 0.42) | <0.001 |

**Table** **S7.** Adjusted Cox regression models for 30-day mortality in 689,197 adults infected by SARS-CoV-2 stratified by diagnosis period

|  | **Mortality within 30 days** | | | |
| --- | --- | --- | --- | --- |
| **N=689,197**  **(events=29,131)** | **Diagnosed before 1st July 2020**  **(n=87,512, events=14,384)** | | **Diagnosed after 1st July 2020**  **(n=601,685, events=14,747)** | |
| **Parameter** | **HR**  **(99%CI)** | **p-value** | **HR**  **(99%CI)** | **p-value** |
| FI (per 0.1-point increment) | 1.24  (1.21 – 1.28) | <0.001 | 1.61  (1.58 – 1.65) | <0.001 |
| Male (Yes vs No) | 2.74  (2.62 – 2.87) | <0.001 | 2.26  (2.16 – 2.36) | <0.001 |
| Age (years) | 1.06  (1.06 – 1.06) | <0.001 | 1.11  (1.11 – 1.12) | <0.001 |
